# Loss of cerebellar neurons in the progression of lentiviral disease: effects of CNS-permeant antiretroviral therapy

**DOI:** 10.1186/s12974-016-0726-0

**Published:** 2016-10-14

**Authors:** Christian Wächter, Lee E. Eiden, Nedye Naumann, Candan Depboylu, Eberhard Weihe

**Affiliations:** 1 Molecular Neuroscience, Institute of Anatomy and Cell Biology, Philipps University Marburg, Robert-Koch-Str. 8, 35032 Marburg, Germany; 2 Section on Molecular Neuroscience, Laboratory of Cellular and Molecular Regulation, National Institute of Mental Health, NIH, Bethesda, MD USA; 3 Experimental Neurology, Department of Neurology, Philipps University Marburg, Marburg, Germany

**Keywords:** Human immunodeficiency virus (HIV), Neuro-AIDS, Dementia, Neurodegeneration, Antiretroviral therapy

## Abstract

**Background:**

The majority of investigations on HIV-associated neurocognitive disorders (HAND) neglect the cerebellum in spite of emerging evidence for its role in higher cognitive functions and dysfunctions in common neurodegenerative diseases.

**Methods:**

We systematically investigated the molecular and cellular responses of the cerebellum as contributors to lentiviral infection-induced neurodegeneration, in the simian immunodeficiency virus (SIV)-infected rhesus macaque model for HIV infection and HAND. Four cohorts of animals were studied: non-infected controls, SIV-infected asymptomatic animals, and SIV-infected AIDS-diseased animals with and without brain-permeant antiretroviral treatment. The antiretroviral utilized was 6-chloro-2′,3′-dideoxyguanosine (6-Cl-ddG), a CNS-permeable nucleoside reverse transcriptase inhibitor. Quantitation of granule cells and Purkinje cells, of an established biomarker of SIV infection (*gp41*), of microglial/monocyte/macrophage markers (IBA-1, CD68, CD163), and of the astroglial marker (GFAP) were used to reveal cell-specific cerebellar responses to lentiviral infection and antiretroviral therapy (ART). The macromolecular integrity of the blood brain barrier was tested by albumin immunohistochemistry.

**Results:**

Productive CNS infection was observed in the symptomatic stage of disease, and correlated with extensive microglial/macrophage and astrocyte activation, and widespread macromolecular blood brain barrier defects. Signs of productive infection, and inflammation, were reversed upon treatment with 6-Cl-ddG, except for a residual low-grade activation of microglial cells and astrocytes. There was an extensive loss of granule cells in the SIV-infected asymptomatic cohort, which was further increased in the symptomatic stage of the disease and persisted after 6-Cl-ddG (administered after the onset of symptoms of AIDS). In the symptomatic stage, Purkinje cell density was reduced. Purkinje cell loss was likewise unaffected by 6-Cl-ddG treatment at this time.

**Conclusions:**

Our findings suggest that neurodegenerative mechanisms are triggered by SIV infection early in the disease process, i. e., preceding large-scale cerebellar productive infection and marked neuroinflammation. These affect primarily granule cells early in disease, with later involvement of Purkinje cells, indicating differential vulnerability of the two neuronal populations. The results presented here indicate a role for the cerebellum in neuro-AIDS. They also support the conclusion that, in order to attenuate the development of motor and cognitive dysfunctions in HIV-positive individuals, CNS-permeant antiretroviral therapy combined with anti-inflammatory and neuroprotective treatment is indicated even before overt signs of CNS inflammation occur.

**Electronic supplementary material:**

The online version of this article (doi:10.1186/s12974-016-0726-0) contains supplementary material, which is available to authorized users.

## Background

More than three decades after the discovery of the human immunodeficiency virus (HIV), HIV infections and their consequences remain a major global health concern. Modern antiretroviral therapy (ART) durably suppresses viral replication, improves immune function, and halts clinical disease progression, yet the prevalence of neurological disorders remains persistently high in HIV^+^ individuals [1]. These variegated neurological complications are grouped under the term HIV-associated neurocognitive disorders (HAND). Clinical features of HAND include cognitive deficits in attention, memory, executive function, and informational processing and also particularly cerebellum-associated motor symptoms including gait disturbance, limb weakness, and tremor [2–4]. Infected individuals also frequently exhibit severe affective disturbances early in the course of disease [5, 6]. The nosology for HAND delineates three levels of severity: HIV-associated asymptomatic neurocognitive impairment (ANI), HIV-associated mild neurocognitive disorders (MND), and HIV-associated dementia (HAD) [7]. While the most severe manifestation of HAND (HAD) is rare in the era of ART, the prevalence of cognitive deficits overall remains high, even among aviremic patients [1, 8, 9]. ANI and MND significantly interfere with activities of daily life (e. g., employment, medication management, etc.) and are associated with an increased risk for early development of symptomatic HAND and even increased mortality compared with neuropsychologically unimpaired HIV^+^ individuals [10–13]. According to widely recognized theories, neuronal damage is mainly caused by the virus-dependent activation of macrophages and microglia. This alters the production of chemokines and cytokines and leads to a dysregulation of immune functions and production of neurotoxic substances and harmful viral proteins in the brain [14–18]. Previous neuropathological studies of HIV infection focused mainly on neocortical brain areas traditionally associated with higher cognitive functions, including frontal, prefrontal, parietal, and temporal cortices and basal ganglia [19–22]. However, there is also emerging evidence for a role of the cerebellum in higher cognitive functions and more recently for an involvement in HAND [23]. In fact, the observation that the cerebellar dentate nucleus is expanded in anthropoid apes and humans in parallel with the prefrontal cortex relative to other species has laid foundations for subsequent investigations revealing an emerging role of the cerebellum in many non-motor as well as motor functions [24]. Thus, a multitude of functional imaging and anatomical and clinical studies support the participation of the cerebellum in cognitive functions such as language, visual-spatial, executive, and working-memory processes [25–28]. Schmahmann and Sherman have promulgated a distinct term for disturbances related to cerebellar damage — the cerebellar cognitive affective syndrome (CCAS) [29]. Comparing the symptoms of CCAS and HAND, there is striking congruency. Unexplained cerebellar atrophy has been found with neuroimaging in HIV patients indicating that neurodegeneration in the cerebellum occurs as a consequence of HIV infection [30]. However, it is unexplored which type of cerebellar neurons degenerate and to what extent neuronal cerebellar degeneration is related to neuroinflammatory events within the cerebellum [31].

Considering this background, the present study systematically investigates for the first time the molecular and cellular patterns of the cerebellar response to lentiviral infection, and the consequences of antiretroviral treatment, in a primate model of HIV infection, the simian immunodeficiency virus (SIV)-infected rhesus macaque.

## Methods

### Virus stock, inoculation procedures, antiretroviral treatment

All experiments involving the use of rhesus macaques were approved by the Animal Care and Use Committee of Bioqual, Inc., and were carried out under the ethical guidelines in the NIH Guide for the Care and Use of Laboratory Animals and adhere to the ARRIVE guidelines. In brief, healthy juvenile male rhesus macaques were inoculated intravenously with ten rhesus infectious doses of cell-free human peripheral blood mononuclear cell (hPBMC)-grown SIV strain deltaB670 and then monitored and examined for clinical evidence of disease [32]. At the time of death, seven animals exhibited clinical signs of AIDS (SIV/+AIDS) and five did not (SIV/-AIDS). Four age-matched non-infected macaques served as controls (Ctrl). In addition, six SIV-infected macaques were treated with 6-chloro-2′,3′-dideoxyguanosine (6-Cl-ddG) when they exhibited clinical signs of AIDS, coincident with a viral load greater than 100,000 virions/mL in plasma and greater than 100 virions/mL in CSF (SIV/+AIDS/+ddG) [32]. Initially, five macaques received daily 10 mg/kg subcutaneously of 2′,3′-dideoxyinosine for 3 weeks for clinical stabilization and then 75 mg/kg per day of 6-Cl-ddG for 6 weeks. Only one macaque was treated daily with 6-Cl-ddG alone (75 mg/kg) for 3 weeks. Table 1 shows the infection status, treatment regime, and clinical status of the macaques used in the present study.

**Table 1:** Infection status, treatment regime, and clinical status at time of death and necropsy of the macaques used

Group	Monkey number	SIV deltaB670 inoculation	AIDS^a^	ART^b^
Control	44	No	No	No
	50	No	No	No
	69	No	No	No
	87	No	No	No
	88	No	No	No
SIV/-AIDS	75	Yes	No	No
	80	Yes	No	No
	85	Yes	No	No
	92	Yes	No	No
	93	Yes	No	No
SIV/+AIDS	71	Yes	Yes	No
	74	Yes	Yes	No
	78	Yes	Yes	No
	79	Yes	Yes	No
	82	Yes	Yes	No
	86	Yes	Yes	No
SIV/+AIDS/+6-Cl-ddG	70	Yes	Yes	Yes
	72	Yes	Yes	Yes
	73	Yes	Yes	Yes
	76	Yes	Yes	Yes
	77	Yes	Yes	Yes
	83	Yes	Yes	Yes
	89	Yes	Yes	Yes^c^
	91	Yes	Yes	Yes

^a^Clinical manifestation of acquired immunodeficiency syndrome

^b^Administration of 2,3-ddl (10 mg/kg s. c. daily) for 3 weeks, following 6-Cl-ddG (75 mg/kg s. c. daily) for 6 weeks

^c^Sole administration of 6-Cl-ddG (75 mg/kg s. c. daily) for 3 weeks

### Tissue preparation, histological staining

At the time of death, the macaques were anesthetized with ketamine and transcardially perfused with PBS and formalin/PBS. Tissue specimens were obtained during necropsy, immersion-fixed overnight, and then post-fixed with Bouin-Hollande solution and processed for paraffin embedding. All cerebellar tissue samples were cut into 7-μm-thick sections using a Thermo Scientific HM 325 rotary microtome. Giemsa staining was performed with Giemsa staining solution (Merck, Darmstadt, Germany). Differentiation of red shades was done with acetic acid and of blue shades with isopropanol 70 %. Sections were covered with Eukitt mounting medium (Sigma-Aldrich, Schnelldorf, Germany).

### Immunohistochemistry

In brief, cerebellar tissue sections were deparaffinized, incubated in citrate buffer (pH 6.0) at 92–95 °C for 10 min [32, 33], subsequently permeabilized and blocked for non-specific binding using the Vecta-Lab avidin/biotin blocking kit (Vector Laboratories, Burlingame, USA), and incubated with primary antibodies. Immunolabeling was performed using fluorochrome-conjugated secondary antibodies (1:200; Dianova) or, alternatively, biotinylated secondary antibodies (1:200; Dianova, Hamburg, Germany), standard avidin-biotin-peroxidase technique (1:500; Vectastain Elite ABC kit, Vector Laboratories), and 3,3′-diaminobenzidine (Sigma, Deisenhofen, Germany) with ammonium nickel sulfate (Fluka, Buchs, Switzerland), resulting in dark blue/black staining. For the visualization of two antigens in the same section, double immunostaining was performed. The primary antibodies used are anti-albumin (rabbit, 1:20.000, Cat No A001, DAKO, Hamburg, Germany), anti-CD68 (mouse, 1:1.000, Cat No M0876, DAKO, Hamburg, Germany), anti-CD163 (mouse, 1:2.000, Cat No MCA1853, AbD Serotec, Puchheim, Germany), anti-GFAP (guinea pig, 1:4.000, Cat No GP52, Progen, Heidelberg, Germany), anti-IBA-1 (rabbit, 1:3.000, Cat No 019–19741, WAKO Chemicals, Neuss, Germany), and SIV_mac_251 *gp41* (mouse, 1:4.000, National Institute of Health, AIDS Research and Reference Program, Germantown, USA). Immunostained sections were analyzed and documented with Olympus AX70 (Olympus Optical, Hamburg, Germany) or Zeiss Axio Imager.M2 (Zeiss, Oberkochen, Germany) microscopes.

### Quantitative image analysis

To achieve an unbiased analysis of immunohistochemical and histochemical staining, animal identity and cohort affiliation were blinded for the investigators. To obtain a high level of comparability, every incubated or stained section was evaluated by an experienced investigator for quality of tissue and staining. Instances of deficient staining of tissue sections were excluded from further analysis.

(i) Quantification of viral tissue load: To quantify the viral load in the cerebellar tissues, up to two non-neighboring SIV glycoprotein GP41 (*gp41*)-stained sections per macaque were analyzed. All SIV *gp41* ^*+*^ cells were counted and related to the section area. To determine the section area, images of the sections were obtained and analyzed with ImageJ software (NIH, Bethesda, MD, USA). Multinucleated giant cells (MNGC) were counted as one cell.
(ii) Analysis of anti-IBA-1 staining: To quantify microglial/macrophage responses, two to three ionized calcium-binding adapter molecule 1 (IBA-1)-stained cerebellar sections per macaque were given a neuroinflammatory activity score, based on microglial activity (distinguished by cell shape, ramified vs. amoeboid) and number of SIV-encephalitis hallmarks (MNGC, microglial nodules) per section. Further information regarding the neuroinflammatory activity score is provided in Additional file 1: Table S1.
(iii) Analysis of anti-CD68 staining: To quantify CD68^+^ cells, six images randomly chosen were taken at a ×400 magnification from up to three non-neighboring anti-CD68-stained sections for each macaque. ImageJ cell counter plug-in was used to determine the number of CD68^+^ cells in each image. The location of CD68^+^ cells was considered by distinguishing between perivascular and parenchymal localizations. Perivascular localization was defined as association to Virchow-Robin spaces and parenchymal localization without any association to vessels. Cells located in the pial meninges were not considered for counting. MNGC counted as one cell.
(iv) Analysis of anti-CD163 staining: To quantify CD163^+^ cells, up to three non-neighboring CD163-stained sections per macaque were analyzed. Distinguishing between intravascular, perivascular, and parenchymal localization, all CD163^+^ cells per section were counted. Intravascular localization was defined as an endothelial-attached cell, perivascular localization as associated to Virchow-Robin space, and parenchymal localization without any association to vessels. Cells located in the pial meninges were not considered for counting. MNGC counted as one cell. The determined number of cells was related to the section area.
(v) Analysis of anti-albumin staining: Up to three non-neighboring albumin-stained sections per macaque were semi-quantitatively analyzed with respect to the extravascular presence of albumin.
(vi) Determination of linear Purkinje cell density: To determine the linear Purkinje cell (PC) density, cerebellar tissue sections were Giemsa stained as described above. Seven to ten images of cerebellar cortical areas were randomly taken in ×100 magnification out of three non-neighboring sections for each macaque. From the images taken per animal, six were randomly chosen and analyzed using ImageJ. For measurement of the PC layer length, a freehand line through the center of the PC bodies was drawn and the PC bodies were counted. The data were captured and summed for each macaque.
(vii) Determination of granule cell density: To determine the density of granule cells (GC), cerebellar tissue sections were Giemsa stained as described. Six images of the GC layer areas were randomly taken in ×400 magnification out of two non-neighboring sections for each macaque. The ImageJ cell counter plug-in was used to determine the number of GC in each image. Only GC with identifiable nuclei were counted. The resulting number of GC was related to the pictured area of the GC layer. Non-corresponding areas, such as truncated vessels of neighboring cortical layers, were subtracted. Data were captured and summed for each macaque.

### Statistical analysis

Statistical analysis was done using the GraphPad Prism 5.0 (GraphPad Software, La Jolla, USA) performing one-way ANOVA and Bonferroni’s post-test for multiple comparisons to evaluate statistical differences between the groups. A value of *p* < 0.05 was considered significant.

## Results

Viral load, innate immunity signatures, astrogliosis, and blood brain barrier (BBB) integrity were monitored by analyzing cerebellar tissue sections of non-infected control macaques (control), SIV-infected macaques without AIDS (SIV/-AIDS), and SIV-infected macaques exhibiting AIDS (SIV/+AIDS). An additional group consisted of macaques with symptoms of AIDS, subsequently treated with CNS-permeant antiretroviral therapy (SIV/+AIDS/+6-Cl-ddG).

### Viral load and innate inflammatory signatures in the rhesus macaque cerebellum in early- and late-stage lentiviral infection and effects of antiretroviral treatment with 6-Cl-ddG

**Fig. 1:**
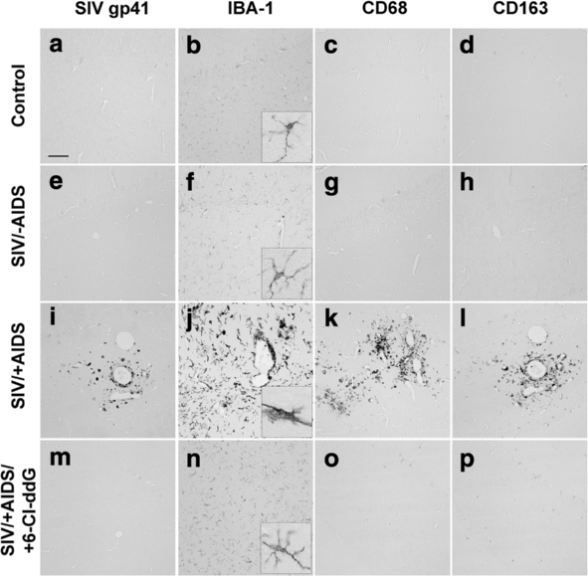
Neuroinflammation. Representative slices stained for SIV *gp41* (**a**–**d**), IBA-1 (**e**–**h**), CD68 (**i**–**l**), and CD163 (**m**–**p**). Note the extensive neuroinflammation in the SIV/+AIDS group (**c**, **g**, **k**, **o**) compared to the Control and SIV/-AIDS groups. SIV^+^ cells are only detectable in the SIV/+AIDS group. Antiretroviral treatment with 6-Cl-ddG lowers the degree of neuroinflammation, but not to base level (**d**, **h**, **l**, **p**). *Scale bar* (in **a**) 100 μm in **a**–**p**, 15 μm in *insets*

**Fig. 2:**
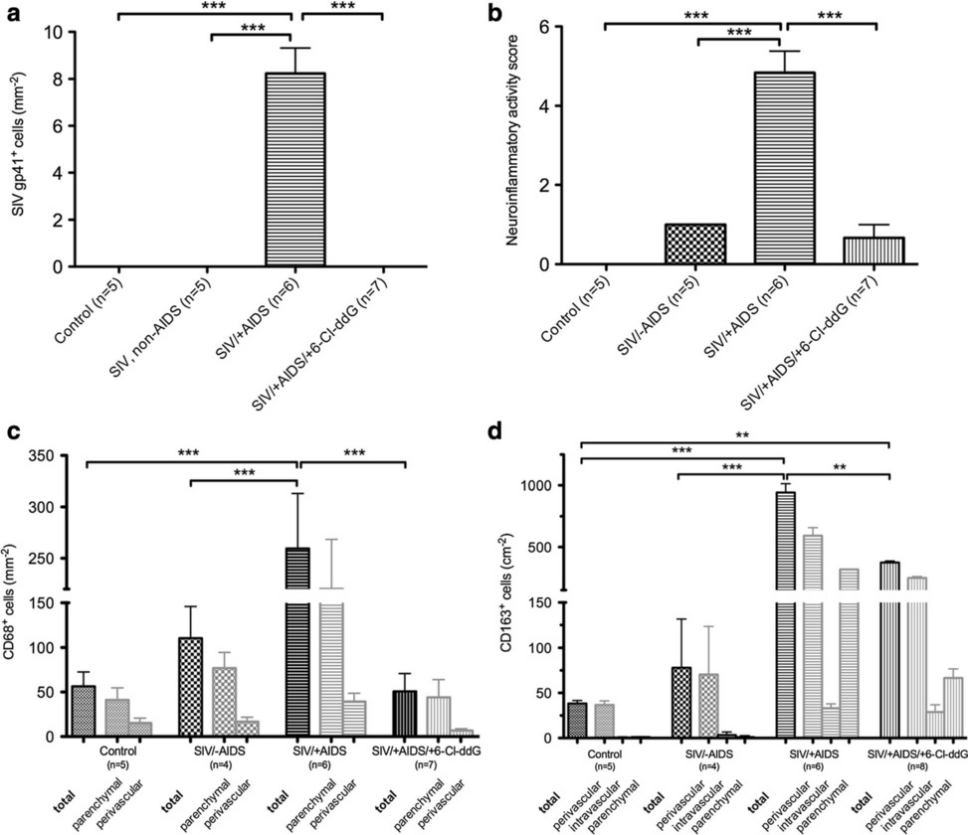
Quantification of SIV *gp41* ^*+*^ (**a**), CD68^+^ (**c**), and CD163^+^ (**d**) cells and neuroinflammatory activity score (**b**). SIV *gp41* ^*+*^ cells were detected only in the cerebellum of the SIV/+AIDS group (8.23 ± 2.16/mm^2^) (**a**). This correlates with a high score in neuroinflammatory activity (4.83 ± 1.33) (**b**). 6-Cl-ddG significantly reduces neuroinflammation (0.67 ± 0.82), but not to base level as seen in the control group (0 ± 0). A subliminal neuroinflammatory activity remains, like in the SIV/-AIDS group (1 ± 0). The number of CD68^+^ cells is significantly increased in the SIV/+AIDS group (259.5 ± 53.7/mm^2^) compared to the control group (56.6 ± 16.2/mm^2^) and the SIV/-AIDS group (110.5 ± 35.4/mm^2^) (**c**). 6-Cl-ddG significantly reduces CD68^+^ cell count to base level (50.8 ± 20.1/mm^2^). CD68^+^ cells show a predominantly parenchymal location. The total number of CD163^+^ cells is increased in the SIV/+AIDS group (943.6 ± 69.5/cm^2^) compared to the control group (38.4 ± 3.3/cm^2^) and the SIV/-AIDS group (80.0 ± 53.5/cm^2^). 6-Cl-ddG reduces significantly the total number of CD163^+^ cells (374.3 ± 12.5/cm^2^), but the number of intravascular and perivascular CD163^+^ cells is constantly high (33.1 ± 4.9 vs. 28.9 ± 8/cm^2^). Illustration of mean values (MV) and standard deviation (SD) (MV ± SD). **p value <0.01, ***p value <0.001

The SIV viral load was determined by immunohistochemistry for SIV *gp41* on the cerebellar tissue sections of the rhesus macaques in each experimental group. SIV *gp41* ^+^ cells were detected only in macaques with AIDS, and not in uninfected control or asymptomatic SIV-infected macaques (Fig. 1a, e, i, m, Fig. 2a). Antiretroviral treatment with 6-Cl-ddG reversed the SIV burden in the cerebellum. Double-staining analysis revealed that SIV *gp41* was expressed in IBA-1^+^ cells, a general marker of cells of monocytic/microglial origin (Additional file 1: Figure S1), but not in astrocytes or neurons.

Using several phenotype markers, we determined the innate immune signature in the cerebellum of macaques during SIV infection and antiretroviral therapy. In comparison to uninfected control macaques, a significant numerical increase of microglia occurred in the cerebellum in the early stage of SIV infection (SIV/-AIDS) as detected with IBA-1. In the late stage of the disease (SIV/+AIDS), signs of SIV-induced inflammation, namely ameboid macrophages, mononuclear nodular infiltrates, and multinucleated giant cells (MNGC), were observed by IBA-1 immunostaining (Fig. 1i, j, k, l, Fig. 2b).

The microglial/macrophage activation markers CD68 and CD163 were occasionally immunodetected in the cerebellum of the control and SIV/-AIDS macaques. Their expression was upregulated in SIV/+AIDS (preferentially in nodule- and giant cell-forming macrophages) (Fig. 1k, l, Fig. 2c, d). CD68^+^ cells were mostly located in the parenchyma whereas CD163^+^ cells were more vessel associated. CD68 expression in this condition likely reflects an M1 macrophage population, whereas CD163 indicates an M2 macrophage phenotype that is thought to represent the trafficking macrophages from the periphery to the brain [34–36].

Antiretroviral treatment with 6-Cl-ddG prevented the appearance of mononuclear infiltrates, mononuclear nodules, and MNGC as detected with IBA-1 and CD68 in SIV/+AIDS (Fig. 1j, k, n, o, Fig. 2b, c). The number of CD163^+^ cells was also reduced by 6-Cl-ddG but was still increased in comparison to control and SIV/-AIDS group (Fig. 1d, h, l, p, Fig. 2d).

### Astroglial reactions in the cerebellum of the rhesus macaques during lentiviral infection and effects of antiretroviral treatment with 6-Cl-ddG

**Fig. 3:**
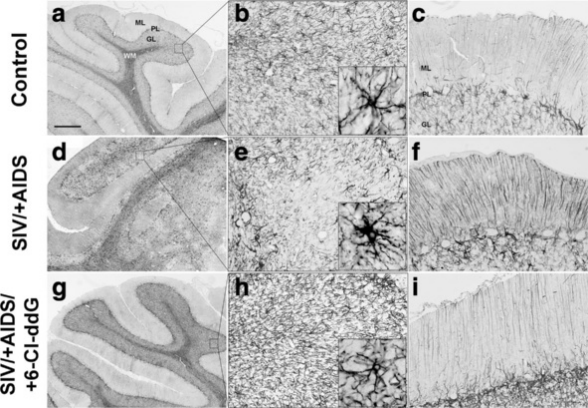
Astrogliosis and Bergmann gliosis. Representative slices stained for GFAP. Note the extensive astrogliosis and Bergmann gliosis in the SIV/+AIDS group (**d**–**f**) with focal rarefication of astroglial structures (**e**). The *inset* in **e** shows a hypertrophic astrocyte. **g**–**i** showing a molecular layer. Note the distinct hypertrophy of Bergmann glia in the SIV/+AIDS group (**f**). 6-Cl-ddG treatment reduces astrogliotic reactions in white matter and molecular layer (**g**–**i**), but not to base level as seen in the control group (**a**–**c**). *Scale bar* (in **a**) 500 μm in **a**, **d**, **g**; 62.5 μm in **b**, **c**, **e**, **f**, **h**, **i**; 20 μm in *insets. ML* molecular layer, *PL* Purkinje layer, *GL* granular layer, *WM* white matter

**Fig. 4:**
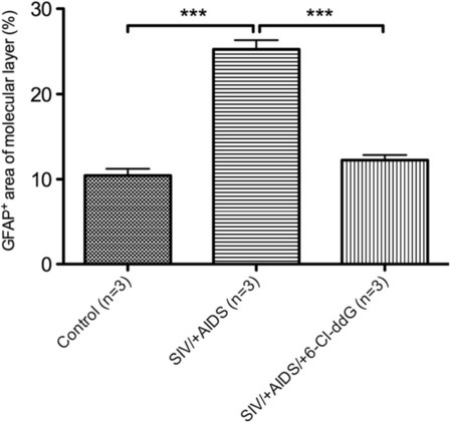
Quantification of GFAP^+^ area of molecular layer. There is a significant increase in GFAP expression in the SIV/+AIDS group (25.24 ± 3.37 %) compared to the control group (10.43 ± 2.55 %). 6-Cl-ddG reduces GFAP expression near to base level as seen in the control group (12.24 ± 1.93 %). Illustration of mean values (MV) and standard deviation (SD) (MV ± SD). ***p < 0.001

Bergmann glia, which are specialized astroglial cells essential for developmental granule cell migration, and positioned with their cell bodies in the vicinity of Purkinje neurons, exhibited increased glial fibrillary acidic protein (GFAP) expression in their processes in the molecular layer in SIV/+AIDS as compared to control macaques (Fig. 3f). Similarly, the astrocytes of the subcortical white matter were also activated and showed upregulated GFAP expression in SIV/+AIDS (Fig. 3d). Both the gliosis of Bergmann glia and cerebellar white matter astrocytes were significantly reduced compared to the SIV/+AIDS group and near to control levels by 6-Cl-ddG (Fig. 3g–i, Fig. 4).

### Perturbation of macromolecular integrity of the BBB in the cerebellum of the rhesus macaques during lentiviral infection and effects of antiretroviral treatment with 6-Cl-ddG

**Fig. 5:**
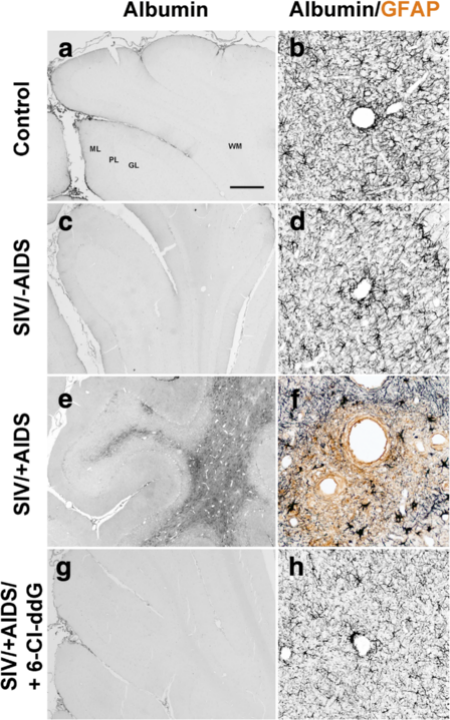
Breakdown of BBB. Representative slices of single (albumin, **a**, **c**, **e**, **g**) and double (albumin/GFAP, **b**, **d**, **f**, **h**) stainings. Note the extensive accumulation of albumin in the parenchyma of the SIV/+AIDS group (**e**) as surrogate for breakdown of the BBB. BBB breakdown correlates with a rarefication of astroglial structures (**f**). Extravascular albumin is undetectable in the other groups. *Scale bar* (in **a**) 500 μm in **a**, **c**, **e**, **g**; 62.5 μm in **b**, **d**, **f**, **h**. *ML* molecular layer, *PL* Purkinje layer, *GL* granular layer, *WM* white matter

In inflammatory foci with mononuclear nodules in the subcortical cerebellar white matter, GFAP^+^ astrocytes were reduced in SIV/+AIDS (Fig. 3d, e, Fig. 5f). In contrast, an increased expression of GFAP in the special astroglial type, the cerebellar Bergmann glia, was detected (Fig. 3f, Fig. 4). Double staining of GFAP with albumin demonstrated that in these foci with reduced GFAP^+^ areas, albumin was accumulated in the parenchyma, suggesting focally restricted macromolecular leakage of the BBB (Fig. 5f). Staining of the adjacent sections for IBA-1 and SIV *gp41* revealed that, in the areas with disintegrity of the BBB, SIV-induced mononuclear nodules, infiltrates, and giant cells occurred (Additional file 1: Figure S2). Antiretroviral treatment with 6-Cl-ddG abolished the viral cerebellar load and the associated productive inflammation, and preserved the macromolecular integrity of the BBB, as monitored by immunohistochemistry for albumin (Fig. 5g, h).

### Neurodegeneration in the cerebellum of the rhesus macaques during lentiviral infection and effects of antiretroviral treatment with 6-Cl-ddG

**Fig. 6:**
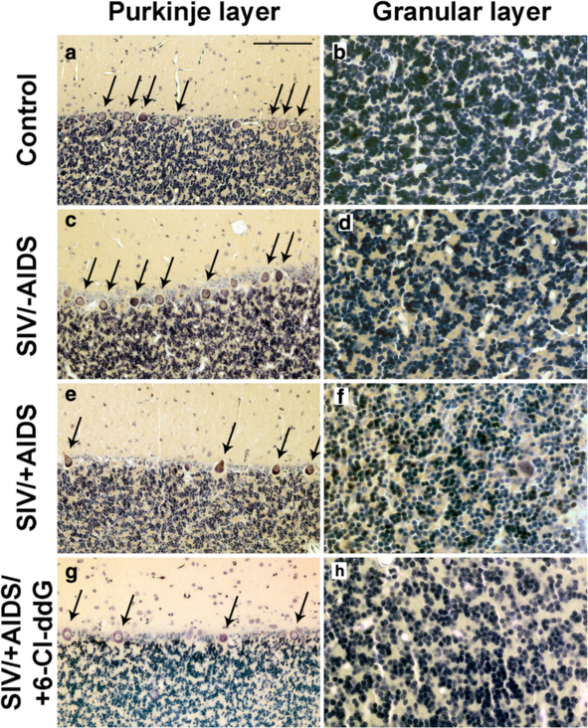
Neurodegeneration. Representative slices of Purkinje layer (**a**, **c**, **e**, **g**) and granular layer (**b**, **d**, **f**, **h**) with Giemsa staining. *Arrows* indicate Purkinje cell bodies. *Scale bar* (in **a**) 100 μm in **a**, **c**, **e**, **g**; 50 μm in **b**, **d**, **f**, **h**

**Fig. 7:**
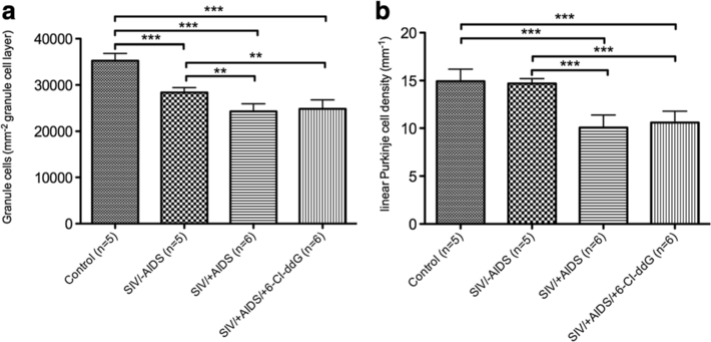
Quantification of neuronal loss. **a** Significant reduction of granule cells (GC) in the SIV/-AIDS group (28,387 ± 1042/mm^2^) and extension in the SIV/+AIDS group (849.9 ± 57.5/0.035 mm^2^) compared to the control group (35,269 ± 1584/mm^2^). 6-Cl-ddG stops the loss of granule cells, but as expected cannot reverse it (24,821 ± 1966/mm^2^). **b** Significant decrease in the linear Purkinje cell density (LPD) in the SIV/+AIDS group (10.07 ± 1.31/mm) compared to the control group (14.93 ± 1.27/mm) and the SIV/-AIDS group (14.69 ± 0.52/mm). 6-Cl-ddG stops, but as expected cannot reverse the reduction in the LPD (10.06 ± 1.19/mm). Illustration of mean value (MV) and standard deviation (SD) (MV ± SD)

The number of granule cells (Giemsa staining) was found to be significantly reduced in SIV/-AIDS as compared to the control (Fig. 6b, d, f, h, Fig. 7a), whereas the linear density of Purkinje neurons was not different from the control in SIV/-AIDS (Fig. 6a, c, e, g, Fig. 7b). In the symptomatic stage, the number of granule cells as well as the linear Purkinje cell density was decreased (Fig. 6e, f, Fig. 7). Antiretroviral treatment with 6-Cl-ddG did not reverse the loss of granule cells or Purkinje neurons in SIV/+AIDS (Fig. 6g, h, Fig. 7).

## Discussion

In the present study, we demonstrate that SIV infection in rhesus macaques causes early neuronal loss of cerebellar granule cells in an asymptomatic stage of disease, characterized by the absence of productive cerebellar infection and neuroinflammatory signs, and late loss of Purkinje cells, correlated with overt infection and inflammation in the cerebellum.

### Vulnerability and resilience of cerebellar neurons in the asymptomatic stage of disease without productive SIV infection

One of the key findings in our study is the significant loss of granule cells as an early event preceding the onset of either clinical signs of SIV disease, marked neuroinflammation, or productive CNS infection. Although early invasion of human CNS by HIV, and primate CNS by SIV, has been reported, CNS inflammation and viral replication appear to be largely absent in the pre-AIDS stage [37, 38]. Previous studies from our own laboratories have demonstrated that neither viral protein nor viral RNA is easily detectable in the non-lymphoid tissues of SIV-infected but asymptomatic macaques [39], compared to later stages of the disease. Bell et al. likewise showed the apparent absence of HIV in the brain in human pre-AIDS cases [40]. Although viral replication in the cerebellum was undetectable in the asymptomatic stage of infection in our study, discrete signs of neuroinflammation, i. e., low-grade microglial and astroglial activation, were seen in the cerebellum during this stage. As the spleen and other lymphoid tissues represent the major reservoir and sites of viral replication in T-lymphocytes and monocytes in asymptomatic animals [41], it is reasonable to assume that early viral effects in the brain are initiated peripherally. Mechanisms for such effects might include the production and release of pro-inflammatory cytokines, in particular tumor necrosis factor alpha (TNF-α) and interleukin 1 (IL-1), from activated peripheral immune cells, leading to the activation of brain-resident microglia and macrophages and the augmentation of brain parenchymal levels of pro-inflammatory cyto- and chemokines [42–45].

In addition, the release of viral proteins in the periphery, with penetrance to CNS, is suspected to impair brain neuronal survival. Crucial roles for the envelope protein glycoprotein GP120 (*gp120)* and the trans-activator of transcription protein (*tat)* in this process have been proposed. Both viral proteins can cross the functional BBB and appear at potentially neurotoxic concentrations in the brain parenchyma [46–48]. Like TNF-α and IL-1, the viral proteins *gp120* and *tat* may exert their neurotoxicity via activation of glutamate (e. g., NMDA) receptors [45, 49–51], leading to dysregulation of calcium homeostasis, overactivation of calcium-dependent signalling pathways, and subsequent neurotoxicity [52].

**Fig. 8:**
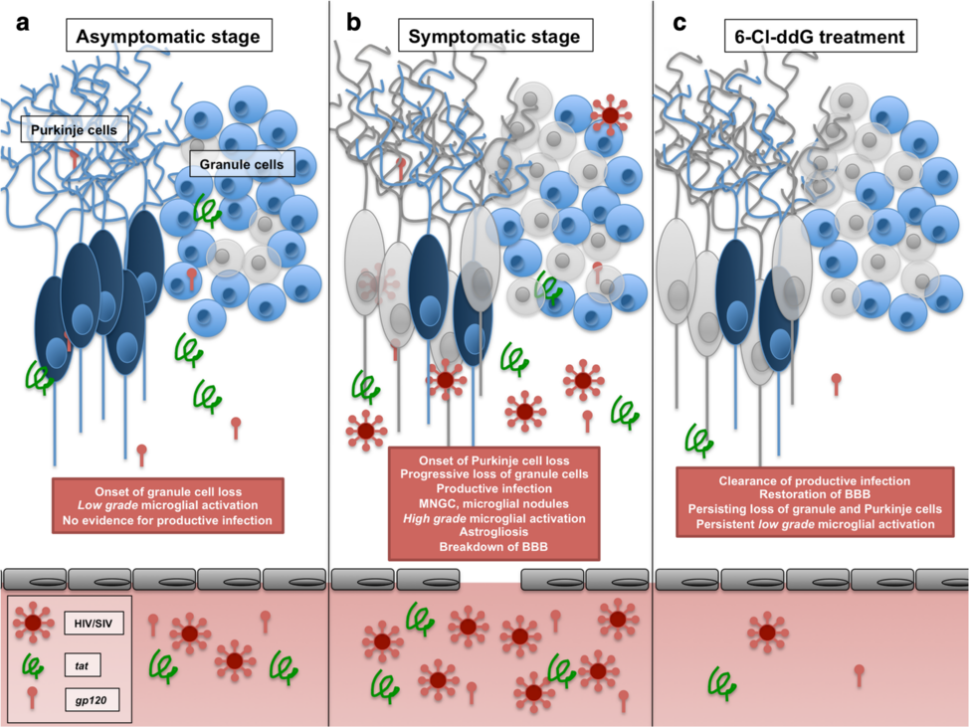
Schematic diagram summarizing the pathophysiological scenarios in the cerebellum in the course of SIV infection and antiretroviral treatment. While productive viral replication is undetectable, low-grade microglial activation is seen in the asymptomatic stage of disease (SIV/-AIDS group, subfigure **a**): *tat* and *gp120* are depicted as presumptive contributors to this process, and granule cell loss is depicted as the result of the collective effects of microglial activation and SIV protein*.* In the symptomatic stage of disease (SIV/+AIDS group, subfigure **b**), productive infection is paralleled by high-grade microglial activation, astrogliosis, and macromolecular defects of the BBB resulting in a further loss of granule cells and onset of loss of Purkinje cells. Antiretroviral treatment with the highly CNS-permeant substance 6-Cl-ddG (SIV/+AIDS/+6-Cl-ddG, subfigure **c**) results in clearance of productive viral infection and restoration of the disintegrated BBB but persistence of low-grade microglial activation and losses of granule cells and Purkinje cells. *MNGC* multinucleated giant cells, *BBB* blood brain barrier

Purkinje cells, unlike granule cells, were resistant to destruction in the early stages of SIV disease. A potential factor in the relative resistance of Purkinje cells is their expression of calbindin, a cytosolic calcium-binding protein, which may protect them, at least temporarily, from the fatal consequences of NMDA-receptor overactivation. Consistent with this hypothesis, Mattson et al. showed an increased resistance of calbindin-expressing neurons, relative to calbindin-negative neurons, against calcium influx-induced neurotoxicity in culture [53]. Ultimately, however, Purkinje cell loss does occur in late-stage SIV disease. A previous study provided evidence for possible Purkinje cell damage in HIV encephalitis by demonstrating the disruption of neurofilament signatures in distinct cerebellar Purkinje cell populations [21]. Excitotoxicity plays a central role also in other neurodegenerative diseases, notably Parkinson’s and Alzheimer’s diseases, and calbindin-expressing neurons seem to be relatively resistant under these conditions [54–56]. Given the relative resistance of calbindin-expressing cells in these diseases, the effects of viral proteins, and virus-induced cytokines and chemokines, may play a specific role in the loss of Purkinje cells in lentiviral encephalitis, compared to these other progressive neurodegenerative diseases. The suspected mechanisms of cerebellar neurodegeneration in the asymptomatic disease stage are illustrated in Fig. 8a.

### Persistence of neurodegeneration in the symptomatic stage of disease with productive SIV infection in the cerebellum

With progression to AIDS, the recognized SIV-encephalitis characteristics can be observed in the cerebellum, according to the early findings of Lackner et al. [57]. High inflammatory and viral load presents in high numbers of productively infected monocytic cells (*gp41*). High levels of inflammation are seen in strong microglial and monocytic cell activation (CD68, IBA-1, CD163), gliosis of astrocytic cell population (Bergmann gliosis and astrogliosis), and breakdown of the BBB, as demonstrated in the present study. These overt neuroinflammatory events in the cerebellum are most likely to enhance progression of the already-existing granule cell loss, and override the intrinsic calbindin-dependent resilience of the Purkinje cells, resulting ultimately to a significant loss in both cell populations. The presumed multifactorial neuroinflammatory factors of neurodegeneration in the symptomatic stage are illustrated in Fig. 8b.

In contrast to the early beginning and progressive loss of cerebellar neurons demonstrated here, our previous studies in the telencephalon of SIV-infected rhesus macaque brain revealed lentivirus-induced neurochemical damage including reduced density of projections of cholinergic basal forebrain neurons to the frontal, parietal, and hippocampal-entorhinal cortex but without changes in the number of these projection neurons [33]. In contrast, degeneration of neocortical large pyramidal neurons and of distinct neuronal populations of the basal ganglia in addition to synaptodendritic damage has been demonstrated [19, 58–62]. Besides these previously recognized neurochemical and neurostructural changes in neocortical areas and basal ganglia, the neuroinflammation-related loss of granule cells and Purkinje cells as shown in the present study is a new finding and may represent an additional nexus for the development of distinct HIV-related motor and cognitive impairments. The occurrence of distinct behavioral impairments has been already demonstrated in a previous study of rhesus macaques infected with the same macrophage tropic SIV strain deltaB670 as used in the present study [63].

One important aim of our study was to establish whether or not decreasing viral load by treatment with brain-permeant antiretroviral therapy late in the disease process would affect neurodegeneration and when in fact neurodegeneration was likely to occur within the larger context of lentiviral disease progression*.* An important logical extension of the work reported here would be earlier treatment with brain-permeant ART and its effects on both neuropathology and behavioral impairments (see Ref. [63]) in SIV disease, as a prelude to consideration of brain-permeant ART as an important adjunct to HAART regimens currently in use in treatment of human HIV disease.

To the best of our knowledge, a significant loss of cerebellar granule cells associated with lentiviral infections has only been shown in cell culture [49, 64]. Apart from case reports, Purkinje cell loss in this context has not been systematically investigated yet [65, 66]. Damage to Purkinje cells plays an important role in the pathophysiology of other neurological disorders affecting the cerebellum like essential tremor or spinocerebellar ataxia [67–69]. Likewise, a significant loss of cerebellar granule and Purkinje cells in Alzheimer’s disease seems to correlate with an advanced stage of disease [70]. Recently, a strong association of distinct and circumscribed cerebellar atrophy with frontotemporal dementia and Alzheimer’s disease and an involvement of the cerebellum in Parkinson’s disease has been shown [71, 72]. Our results support these observations, suggesting that the cerebellum may be an important albeit as-yet unrecognized brain participant in dementia and cognitive dysfunction associated with major neurodegenerative diseases. As numerous behavioral studies have indicated that the SIV model resembles the neurobehavioral aspects of HIV disease in HIV-infected patients [73], further attention to cerebellar neuropathology and dysfunction may be warranted in investigations of the CNS aspects of human HIV infection and chronic disease.

### The impact of brain-permeant antiretroviral therapy

In concordance to our previous results and the findings of Fujii et al., the highly lipophilic antiretroviral drug 6-Cl-ddG was found to effectively inhibit SIV replication in the cerebellum [32, 74]. Although 6-Cl-ddG significantly reduced the brain viral load and consecutively eliminated productive SIV infection in the cerebellum, a low-grade microglial/macrophage activation is persistent as indicated by the constantly high number of perivascular CD163^+^ cells. This suggests an increased trafficking of immune cells from the periphery into the brain and possibly a switch in the polarization of macrophage phenotypes to an anti-inflammatory subtype of macrophages. Ongoing persistent neuroinflammation under ART recently revealed by modern in vivo PET technique is in good accordance with our histopathological findings and has been shown to correlate with poorer executive performance [75]. The consequences of antiretroviral therapy are illustrated in Fig. 8c.

## Conclusions

We provide evidence that in SIV infection of the rhesus macaque, neuronal cell loss in the cerebellum precedes high viral load and peak levels of inflammation and continues as the viral load and inflammation increases during progression to AIDS. Treatment with a brain-permeant antiretroviral during the symptomatic stage of viral disease is highly effective, and prompt, to reduce the viral load concomitantly with the inflammatory load. However, and as expected, severe neuronal loss is not reversed by this treatment, suggesting that only early initiation of brain-permeant antiretroviral therapy is likely to prevent neuronal loss in the cerebellum and elsewhere in the brain. The present results, insofar as the SIV-infected rhesus macaque is an informative model for human HIV infection, support current guidelines to initiate antiretroviral therapy soon after evidence of infection, and independently of peripheral signs and symptoms of disease progression, including CD4^+^ cell loss and increasing intensity of viral load. In fact, our results suggest that with respect to early granule cell loss, brain-permeant antiretroviral treatment can be avoided altogether, provided that ART is initiated sufficiently early in the course of the disease to prevent the entry of neurotoxic protein products of peripheral infection into the brain.

## Additional file

Additional file 1:
**Figure S1.** Colocalization of IBA-1/SIV *gp41*. **Figure S2.** Breakdown of BBB. **Table S1.** Neuroinflammatory activity score. PDF 526 kb)

## Abbreviations

ANI: Asymptomatic neurocognitive impairment
ART: Antiretroviral therapy
BBB: Blood brain barrier
CCAS: Cerebellar cognitive affective syndrome
CD: Cluster of differentiation
GFAP: Glial fibrillary acidic protein
*gp41*: Glycoprotein GP41
HAD: HIV-associated dementia
HAND: HIV-associated neurocognitive disorders
IBA-1: Ionized calcium-binding adapter molecule 1
MND: Mild neurocognitive disorder
