# Supplementary material for: Loss of cerebellar neurons in the progression of lentiviral disease: effects of CNS-permeant antiretroviral therapy
Source: J Neuroinflammation. 2016 Oct 14;13:272. doi: 10.1186/s12974-016-0726-0 (PMC5064958; doi:10.1186/s12974-016-0726-0)
Supplement: Additional file 1: — Figure S1. Colocalization of IBA-1/SIV gp41. Figure S2. Breakdown of BBB. Table S1. Neuroinflammatory activity score. PDF 526 kb) [file 12974_2016_726_MOESM1_ESM.pdf]

SUPPLEMENTARY MATERIAL

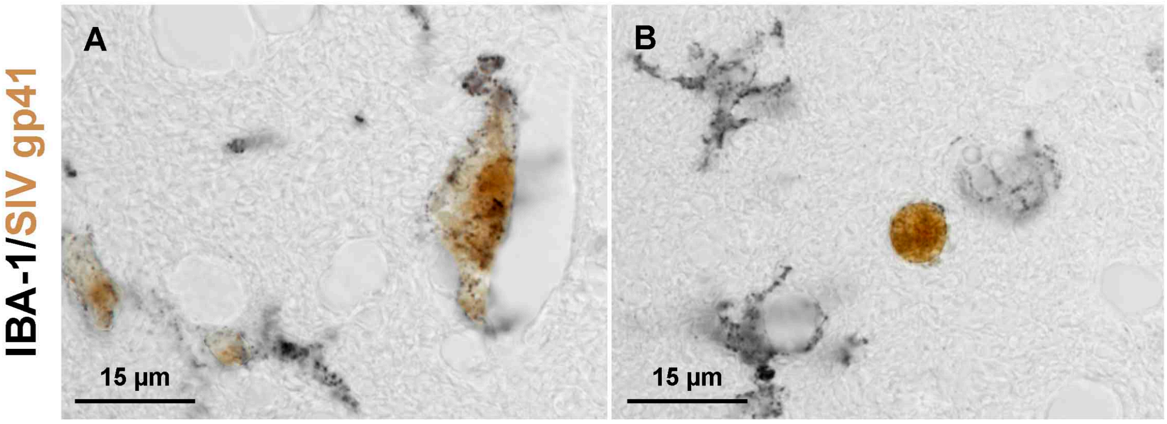

Supplementary figure 1 Colocalization of IBA-1/SIV *gp41*. SIV *gp41*$^{+}$ cells are of monocytic origin, visualised with IBA-1.

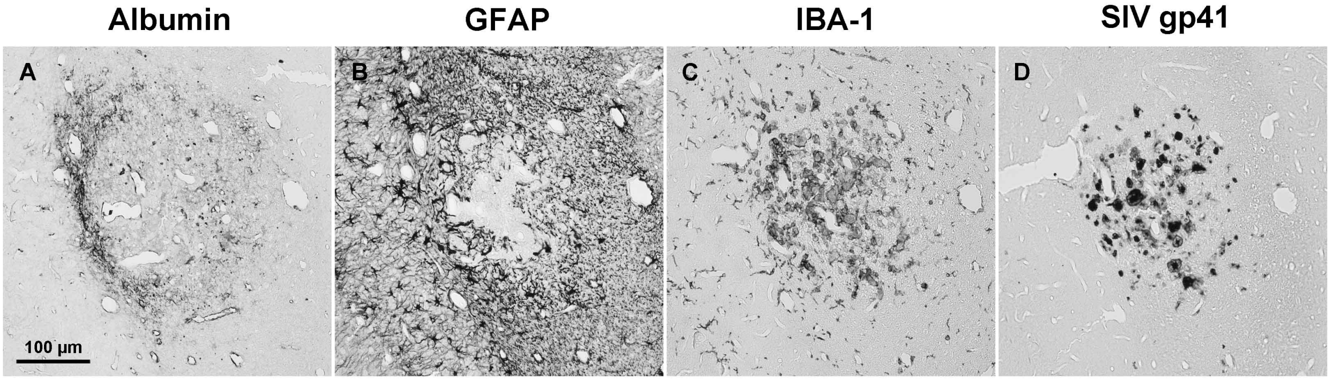

Supplementary figure 2 Breakdown of BBB. Consecutive sections showing macromolecular breakdown of BBB. Parenchymal albumin accumulation (A) is associated with rarefication of astrocytic structures (B) and accumulation of infected monocytic cells (C, D).

Supplementary table 1 Neuroinflammatory activity score. Reaction of IBA-1^{+} cells of monocytic origin in the cerebellum during SIV infection. Features like cell shape (ramified vs. ameboid), dissemination (focally vs. diffuse) and density of multinucleated giant cells (MNGC) and microglial nodules were visualised with IBA-1, scored and summed up.

	**Neuroinflammatory activity score**									
	**Cell shape**			**MNGC**			**Microglial nodules**			**Total**
	ramified	ameboid		none	$<5/cm^2$	$>5/cm^2$	none	$<3/cm^2$	$>3/cm^2$	**0-6**
		focally	diffuse							
**Score**	**0**	**1**	**2**	**0**	**1**	**2**	**0**	**1**	**2**	
